# High level of interleukin-32 gamma in the joint of ankylosing spondylitis is associated with osteoblast differentiation

**DOI:** 10.1186/s13075-015-0870-4

**Published:** 2015-12-04

**Authors:** Eun-Ju Lee, Eun-Jin Lee, Yeon-Ho Chung, Da-Hyun Song, Seokchan Hong, Chang-Keun Lee, Bin Yoo, Tae-Hwan Kim, Ye-Soo Park, Soo-Hyun Kim, Eun-Ju Chang, Yong-Gil Kim

**Affiliations:** Department of Rheumatology, University of Ulsan College of Medicine, Asan Medical Center, 88 Olympic-ro 43 gil, Songpa-gu, Seoul, 05505 Korea; Department of Biomedical Sciences, Cell Dysfunction Research Center and BMIT, University of Ulsan College of Medicine, Asan Medical Center, 88 Olympic-ro 43 gil, Songpa-gu, Seoul, 05505 Korea; Department of Rheumatology, Hanyang University Hospital for Rheumatic Diseases, Seoul, 133-791 Korea; Department of Orthopaedic Surgery, Guri Hospital, Hanyang University College of Medicine, Kyunggi-do, 471-701 Korea; Department of Biomedical Science and Technology, Konkuk University, Seoul, 143-701 Korea

**Keywords:** Interleukin-32, Osteoblast differentiation, Ankylosing spondylitis

## Abstract

**Backgound:**

The formation of bony spurs and ankylosis is a key pathognomic feature in ankylosing spondylitis (AS) and results in functional impairment. The aim of this study was to investigate the role of IL-32γ in osteoblast (OB) differentiation and its association with the pathogenesis of AS.

**Methods:**

The concentration and expression of IL-32γ were evaluated in synovial fluid and tissue from patients with AS, rheumatoid arthritis (RA) and osteoarthritis (OA), using enzyme-linked immunosorbent assay and immunohistochemistry. To establish whether IL-32γ affects OB differentiation, we used calvarial cells of *IL-32γ* transgenic (TG) mice or wild-type (WT) mice. To elucidate the mechanism of osteoblastogenesis, levels of regulators were assayed in *IL-32γ* TG mice and in primary OBs after IL-32γ stimulation.

**Results:**

The IL-32γ levels were higher in the synovial fluid of AS patients compared with RA or OA patients and the expression of IL-32 was higher in AS synovia than in RA or OA synovia. Additional IL-32γ stimulation in precursor cells enhanced OB differentiation potentially and *IL-32γ* TG mice showed higher rates of OB differentiation than WT mice. IL-32γ reduced the expression of DKK-1, a negative regulator, in both WT precursor cells and human OBs and the constitutive expression of DKK-1 was suppressed in calvarial cells from *IL-32γ* TG mice.

**Conclusions:**

The elevated level of IL-32γ in AS joint could enhance OB differentiation via DKK-1 suppression. Therefore, IL-32γ might be a putative molecular target to prevent the abnormal bone formation in AS.

**Electronic supplementary material:**

The online version of this article (doi:10.1186/s13075-015-0870-4) contains supplementary material, which is available to authorized users.

## Backgound

Ankylosing spondylitis (AS) is a chronic inflammatory form of arthritis that primarily affects the spine. The formation of bony spurs and ankylosis, which cause functional impairment in AS patients, are the characteristic axial findings of AS. Besides the axial skeleton, peripheral joints are also involved in about 30–50 % of primary AS during the disease course, which is usually follows an asymmetric and oligoarticular pattern [1, 2]. Interestingly, new bone formation at entheseal sites of the peripheral joint was commonly seen in AS [3].

The new bone formation in AS might be related to active repair following the damage caused by inflammation [4, 5]; that is, inflammation and bone formation appear to occur at different times, with the former preceding the latter. However, direct bone damage does not seem to be essential, given that inhibition of the activity of the osteoclasts did not prevent bone formation in animal experiments [6]. Therefore, the mechanism that underlies the proliferative bone formation in AS remain uncertain.

Bone formation requires the differentiation and activation of osteoblasts (OBs), which synthesize the bone matrix and originate from mesenchymal stem cells. The differentiation and activation of OBs are regulated by various molecules. Of these, parathyroid hormone, bone morphogenetic protein (BMP), and members of the Wnt protein family are the most prominent factors. Wnt proteins activate at least three distinct pathways: the canonical (β-catenin-dependent), calcium-dependent, and planar polarity pathways [7]. Of these, the canonical pathway is best understood. Briefly, when Wnt molecules bind to Frizzled and Lrp5/6 on the surface of osteoprogenitors, stabilized β-catenin translocates into the nucleus and enhances the transcription of genes related to OB formation [8]. Dickkopf-1 (DKK-1) is a product of OBs that is a potent Wnt pathway inhibitor and inhibits proper differentiation of OBs. Mice that overexpress DKK-1 in OBs develop osteopenia because of reduced OB abundance and decreased bone formation [9]. Blocking DKK-1 transforms the bone-destruction pattern to a bone-creation pattern in a mouse model [10].

Interleukin (IL)-32, which exhibits the properties of a proinflammatory cytokine, is produced by T lymphocytes, natural killer cells, epithelial cells, blood monocytes and fibroblast-like synoviocytes in the joints and affects various inflammatory cascades [11–13]. Previously, we reported that IL-32γ, the biologically active isoform of IL-32, is a potent mediator of osteoclast differentiation [14, 15]. However, the biologic function of IL-32γ on OBs, the other side of bone balance, has never been investigated.

We report here that IL-32γ accumulates in the joint fluid and is expressed in the synovia of AS patients at much higher levels than in the synovia of rheumatoid arthritis (RA) patients. We first investigated the potentially pathogenic role of IL-32γ in AS, focusing on OB differentiation using *IL-32γ* transgenic (TG) mice.

## Methods

### Sample collection and enzyme-linked immunosorbent assay

All biologic samples from patients were obtained in accordance with the approval of the Asan Medical Center Institutional Review Board (S2013-0986-0003). Informed consent was obtained from all patients. Peripheral synovial tissues (from patients who underwent synovectomy), soft tissues from the axial skeleton (from patients who performed laminectomy) and knee joint fluids were collected at the Asan Medical Center and Hanyang University Hospital. Diagnoses either met the modified New York criteria for AS [16], the 1987 revised criteria for RA [17], or criteria for osteoarthritis (OA) [18]. Clinical information at the time of arthrocentesis was extracted from an electronic clinical database. The concentrations of IL-32γ and tumor necrosis factor (TNF)-α were measured using commercially available kits obtained from YbdY (Seoul, South Korea) and R&D Systems (Minneapolis, MN, USA). A commercial enzyme-linked immunosorbent assay (ELISA) kit (R&D Systems) was also used to determine the murine DKK-1 proteins secreted in culture media.

### Immunohistochemistry assay

Sections of paraffin-embedded synovial tissue samples from OA, RA, and AS patients were stained with anti-IL-32 antibody (Millipore, Billerica, MA, USA) or normal rabbit IgG (Santa Cruz Biotechnology, Dallas, TX, USA) according to manufacturer's instructions. Tissue sections (5 μm thick) were baked at 65 °C for 30 min, and the paraffin was removed by two washes (5 min each) with xylene. The sections were rehydrated by passage through a graded series of ethanol solutions (100 % to 70 % ethanol) and, for antigen retrieval, slides were dipped in citrate buffer (pH 6.0) and incubated at 95 °C for 5 min. For permeabilization, the samples were incubated for 10 min in phosphate-buffered saline (PBS) that contained 0.25 % Triton X-100 (PBST), and were washed twice with PBS, with each wash lasting 5 min. To block nonspecific binding of the antibodies, the samples were incubated with 1 % bovine serum albumin (BSA) in PBST for 1 h and washed twice with PBST, with each wash lasting 5 min. To eliminate endogenous peroxidase activity, the tissue sections were incubated with 3 % H_2_O_2_ in PBS for 30 min and washed twice with PBST, with each wash lasting 5 min. The sections were incubated with anti-IL-32 (1:100 in PBST) or normal rabbit IgG (1:100 in PBST) for 30 min at room temperature, and then washed twice with PBST (with each wash lasting 5 min). Thereafter, the samples were incubated for 30 min at room temperature with anti-mouse or anti-rabbit secondary antibodies that were conjugated to polymeric horseradish peroxidase (Dako, Glostrup, Denmark). For colorimetric detection of the enzymatic reactions, the sections were incubated with Dako liquid DAB+ substrate chromogen solution (Dako) for 30 min, before two washes (5 min each) with PBS. The samples were counterstained with hematoxylin (Sigma, St Louis, MO, USA) for 1 min, and then washed twice with distilled water (each wash for 5 min). To stain the nuclei blue, the slides were dipped once in 0.3 % ammonia in water.

### Animals

*IL-32γ* TG and wild-type (WT) C57BL/6 mice were obtained from Dr. Kim’s laboratory (Konkuk University, Korea) and SLC, Inc. (Japan), respectively. All experiments using mice were performed in accordance with the relevant guidelines and regulations on the use of animals at the Asan Biomedical Research Institute of the Asan Medical Center (Korea) and were approved by the Institutional Animal Care and Use Committee of the Asan Biomedical Research Institute of the Asan Medical Center (2013-02008).

### Osteoblast differentiation

OB differentiation was performed by isolating primary mouse OB precursor cells from the calvariae of 1-day-old mice in accordance with a published protocol [19]. Osteoblastic precursor cells of WT and *IL-32γ* TG mice were isolated from calvaries by six routine sequential digestions with 0.1 % collagenase (Gibco BRL, Gaithersburg, MD, USA) and 0.2 % dispase (Roche, Penzberg, Germany). To induce OB differentiation, these cells were seeded onto 48-well culture plates at a density of 2 × 10^4^ cells/well and cultured in osteogenic medium (α-MEM, 10 % fetal bovine serum (FBS), 10 mM β-glycerophosphate, and 50 mg/ml ascorbic acid) for 1 to 4 weeks. The medium was changed every 3 days. OB differentiation and mineralization were assessed by detecting alkaline phosphatase (ALP) activity or by staining with Alizarin Red (AR) or Von Kossa (VK) stain.

Human OBs were purchased from Promo Cell (Heidelberg, Germany). Cells were cultured in OB growth medium (C-27001) containing fetal calf serum and 100 U/ml of the penicillin-streptomycin at 37 °C in a humidified atmosphere under 5 % (v/v) CO_2_. Human OBs were treated with IL-32γ (50 or 100 ng/mL) for 8 h.

### Reverse transcription-polymerase chain reaction analysis

Total RNA was isolated from the cells using QIAzol Lysis reagent (Qiagen, Valencia, CA, USA) and 2 μg was reverse-transcribed using SuperScript II reverse transcriptase (Life Technologies, Carlsbad, CA, USA) according to manufacturer's instructions. The cDNA generated was amplified by polymerase chain reaction (PCR) using the primers shown in Table 1. The PCR conditions were as follows: denaturation at 94 °C for 30 s, followed by annealing at 55–60 °C for 30 s, and extension at 72 °C for 1 min. The number of cycles fell within the range associated with linear amplification (28–34 cycles; GAPDH required 23 cycles).

**Table 1:** List of primers used for the detection of DKK-1, BMP-2, BMPRII and LRP-5

Target	Sequences
Mouse DKK-1	Forward 5’-GAG GGG AAA TTG AGG AAA GC-3’
	Reverse 5’-GCA GGT GTG GAG CCT AGA AG-3’
Mouse BMP-2,	Forward 5’-GGG ACC CGC TGT CTT CTA GT-3’
	Reverse 5’-TCA ACT CAA ATT CGC TGA GGA C-3’
Mouse BMPRII	Forward 5’-TTG GGA TAG GTG AGA GTC GAA T-3’
	Reverse 5’-TGT TTC ACA AGA TTG ATG TCC CC-3’
Mouse LRP-5	Forward 5’-CAG GTG CTT GTG TGG AGA GA-3’
	Reverse 5’-GTC CAT GAC GAA GTC CAG GT-3’
Mouse GAPDH	Forward 5’-AGC CAC ATC GCTCAG ACA-3’
	Reverse 5’-GCC CAA TAC GAC CAAATC C-3’
Human DKK-1	Forward 5’-CAT CAG ACT GTG CCT CAG GA-3’
	Reverse 5’-CCA CAG TAA CAA CGC TGG AA-3’
Human GAPDH	Forward 5’-CGT CTT CAC CAC CAT GGA GA-3’
	Reverse 5’-CGG CCA TCA CGC CAC AGT TT-3’

*BMP* bone morphogenetic protein, *BMPRII* bone morphogenetic protein receptor II, *DKK-1* Dickkopf-1, *LRP-5* low-density lipoprotein receptor-related protein 5

### Western blotting analysis

WT calvarial OB precursor cells were stimulated with IL-32 (100 ng/ml) and wnt3a (20 ng/ml) in osteogenic media. At the indicated times, the cells were washed with ice-cold PBS and lysed in modified RIPA buffer (50 mM Tris/HCl (pH 7.4), 1 % Nonidet P40, 0.25 % sodium deoxycholate, and 150 mM NaCl) containing protease and phosphatase inhibitors. Cell lysates were centrifuged at 10,000 g for 15 min, the supernatants were collected, and the proteins resolved in 10 % SDS-PAGE gels. Separated proteins were transferred to a polyvinylidene difluoride membrane (Bio-Rad, Hercules, CA USA), and then blocked for 1 h with 5 % BAS (MP biomedicals, Auckland, New Zealand) solution in Tris-buffered saline solution containing 0.1 % Tween 20. The membrane was then incubated overnight at 4 °C with antibodies specific for active β-catenin (Non-phospho, Ser33/37/Thr41, Cell Signaling), total β-catenin, and β-actin, washed, and incubated for 1 h at room temperature with the horseradish peroxidase-conjugated secondary antibody. Reactive proteins were visualized using a chemiluminescence system (Merck-Millipore, Darmatadt, Germany).

### Statistical analysis

The differences between two groups were calculated using the Mann-Whitney *U*-test or an unpaired Student’s *t*-test, and the differences among three groups were analyzed by one-way analysis of variance. The relationships among parameters were tested by using Spearman’s rank correlation coefficient. Statistical analyses were considered significant for *p* values < 0.05.

## Results

### IL-32 in the synovial fluids and tissues

**Fig. 1:**
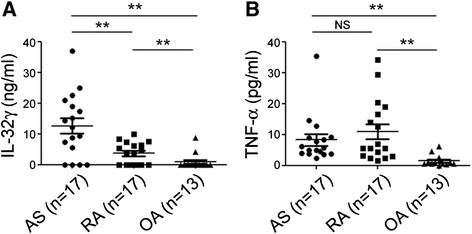
IL-32 in the synovial fluids of ankylosing spondylitis patients. **a** IL-32γ levels in joint fluid and **b** TNF-α levels in joint fluid. Levels of human IL-32γ and TNF-α in the joint fluid of patients with AS (n = 15), RA (n = 17), and OA (n = 13) were measured using a commercially available ELISA kit. The bars show the means ± SD; **p < 0.01. *AS* ankylosing spondylitis, *IL* interleukin, NS not significant, *OA* osteoarthritis, *RA* rheumatoid arthritis, *TNF* tumor necrosis factor

To evaluate the involvement of IL-32 in AS pathogenesis, we first determined the levels of IL-32γ and TNF-α in the joint fluids from patients with AS, RA, and OA. As shown in Fig. 1a, the levels of IL-32γ were significantly higher in AS patients than in either RA or OA patients (*p* < 0.01). The levels of TNF-α in the joint fluids from patients with AS or RA were higher than those from patients with OA. However, there was no difference in the TNF-α level between patients with AS and those with RA (Fig. 1b).

Table 2 summarizes the baseline characteristics of the AS patients (n = 15) and RA patients (n = 17) who underwent arthrocentesis. In the AS patients, none of the clinical parameters, including age, sex, levels of inflammatory markers, grade of sacroiliitis, eye involvement, HLA B27 allele, Bath Ankylosing Spondylitis Disease Activity Index score [20], and modified Stoke Ankylosing Spondylitis Spine Score (mSASSS) [21], was found to be associated with the IL-32γ levels in the peripheral joint fluids (Table 2). Considering mismatch between mSASSS and IL-32 levels of peripheral joint fluid, peripheral arthritis could not reflect the severity of the axial joint directly.

**Table 2:** Baseline characteristics of patients with ankylosing spondylitis and rheumatoid arthritis

	AS (n = 15)	RA (n = 17)	*p*-value
Age (years, mean ± SD)	32.9 ± 9.6	56.2 ± 10.4	<0.0001
Sex (male, n (%))	12 (80.0 %)	2 (11.8 %)	<0.0001
ESR (mm/h, mean ± SD)	53.7 ± 35.5	56.5 ± 31.3	NS
CRP (mg/dL, mean ± SD)	3.5 ± 3.5	2.9 ± 3.4	NS
Disease duration (years, mean ± SD)	5.2 ± 6.1	6.1 ± 6.3	NS
Eye involvement (n (%))	5 (33.3 %)	N/A	
HLA-B27 positive (n (%))	13 (86.7 %)	N/A	
BASDAI (mean ± SD)	4.3 ± 2.3	N/A	
mSASSS (mean ± SD)	15.8 ± 16.7	N/A	
Sacroiliitis grading (n (%))			
Grade 2	3 (20.0 %)	N/A	
Grade 3	9 (60.0 %)	N/A	
Grade 4	3 (20.0 %)	N/A	
Current medications (n (%))			
MTX	7 (46.7 %)	14 (82.4 %)	0.062
SSZ	8 (53.3 %)	1 (5.9 %)	0.005
TNFi	3 (20 %)	1 (5.9 %)	0.319
NSAID	13 (86.7 %)	16 (94.1 %)	0.589

*AS* ankylosing spondylitis, *BASDAI* bath ankylosing spondylitis disease activity index, *CRP* C-reactive protein, *ESR* erythrocyte sedimentation rate, *mSASSS* modified Stoke Ankylosing Spondylitis Spine Score, *MTX* methotrexate, *N/A* not available, *NS* not significant, *NSAID* nonsteroidal anti-inflammatory drug, *RA* rheumatoid arthritis, *SSZ* sulfasalazine, *TNFi* tumor necrosis factor inhibitor

**Fig. 2:**
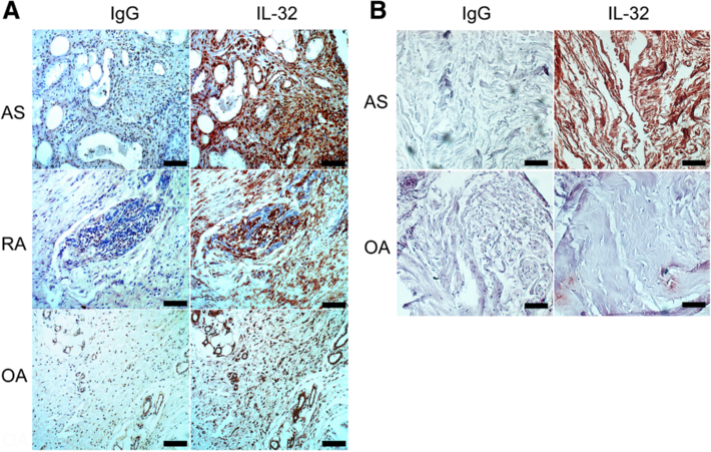
High IL-32 in the peripheral synovia and the axial skeletons of ankylosing spondylitis. **a** Representative immunohistochemical (IHC) images of peripheral synovia of AS, RA, or OA stained with antibodies against IL-32 or isotype controls. Synovitis was found in the tissues acquired from the patients with clinically active AS (36-year-old female, ankle) and RA (52-year-old female, ankle). **b** Representative IHC images of axial skeletons of advanced AS (57-year-old female) and OA (79-year-old female) patients who underwent laminectomy. All IHC images are shown at 200× magnification (scale bars, 100 μm), and are representative of images from three independent experiments. *AS* ankylosing spondylitis, *IL* interleukin, *OA* osteoarthritis *RA*, rheumatoid arthritis

We next performed immunohistochemical (IHC) staining to investigate the levels of IL-32 protein in the joint tissues from patients with AS, RA, and OA. The IHC analysis revealed that the IL-32 level was higher in AS synovial tissues than in RA or OA synovial tissues (Fig. 2a). The stained cells in the synovial tissue possess spindle-shaped morphology and these are mostly consistent with synovial fibroblasts. Further, as shown in Fig. 2b, high IL-32 level was also detected in axial skeletons around the facet joint of AS patients compared to those of OA patients.

### Osteoblast differentiation by IL-32γ is accompanied by the suppression of *DKK-1* expression

**Fig. 3:**
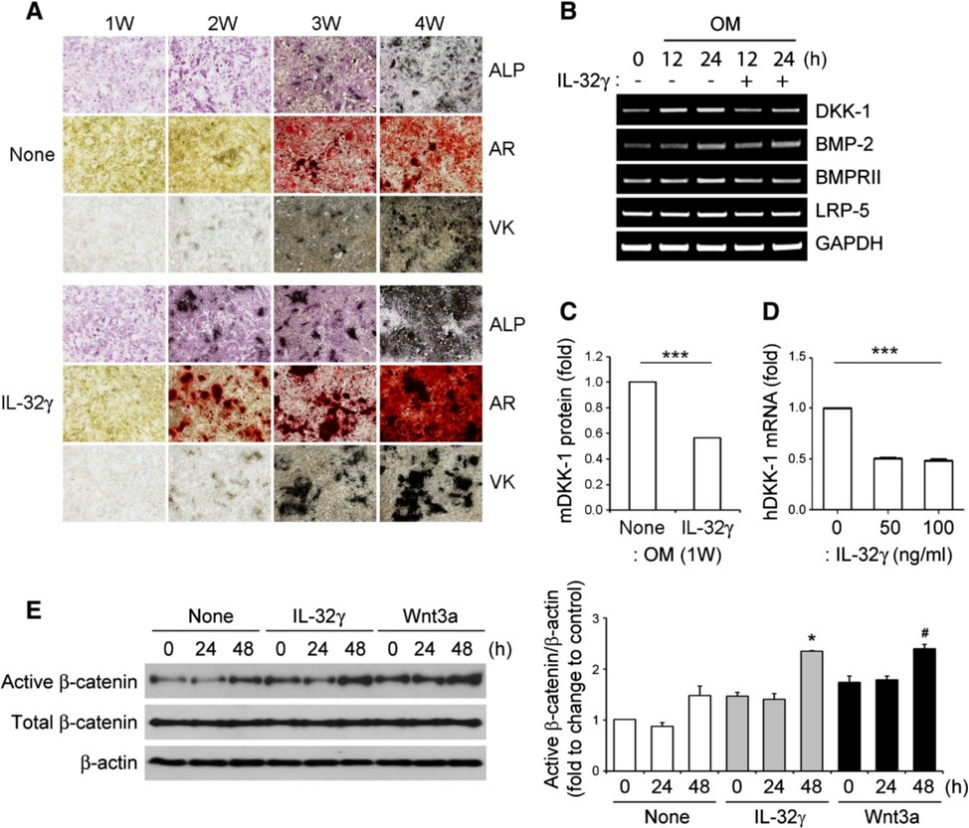
Effect of exogenous IL-32γ on osteoblast differentiation. **a** WT calvarial osteoblast (OB) precursor cells were cultured for 4 weeks with osteogenic medium in the absence (None) or presence of IL-32γ (100 ng/ml) to induce OB differentiation. The cells were fixed once a week and were used for measurement of alkaline phosphatase (*ALP*) or for staining with alizarin red (*AR*) or Von Kossa (*VK*). **b** WT calvarial cells were stimulated with IL-32γ (100 ng/ml) in the presence of osteogenic media (*OM*) for the indicated time and subjected to reverse transcription PCR analysis of the expression of genes that encode *DKK-1*, *BMP-2*, *BMPRII*, and *LRP-5*. **c** The DKK-1 protein level in the culture supernatant from the cells after 1 week of OB differentiation in the absence (None) or presence of IL-32γ was determined by ELISA. **d** The relative expressions of DKK-1 mRNA were evaluated in human OBs treated with IL-32γ (50 and 100 ng/ml). **e** WT calvarial cells were stimulated with IL-32γ (100 ng/ml) and Wnt3a (20 ng/ml) in osteogenic media for the indicated times and whole cell lysates obtained from cultured cells were analyzed by western blotting with antibodies specific for active β-catenin, total β-catenin or β-actin (loading control). The relative protein levels were quantified by densitometry. Data are expressed as the mean ± SD of triplicate experiments; ***p < 0.001, *p < 0.05, versus 0 h treated with IL-32γ; ^#^ *p* < 0.05, versus 0 h treated with Wnt3a. *BMP* bone morphogenetic protein, *BMPRII* bone morphogenetic protein receptor II, *DKK-1* Dickkopf-1, *IL* interleukin, *LRP-5* low-density lipoprotein receptor-related protein 5, *W* week

To understand the direct role of IL-32γ during OB differentiation, calvarial cells of WT mice were treated with IL-32γ (100 ng/ml) in osteogenic media, and changes in ALP activity, calcium deposition (determined by AR staining), and mineral deposition (determined by VK staining) were monitored for 4 weeks. As shown in Fig. 3a, exposure to exogenous IL-32γ promoted rapid and marked OB differentiation and matrix maturation, as indicated by increased ALP activity and increased numbers of AR- and VK-positive cells. Moreover, regions positive for AR staining were observed only after 2 weeks of IL-32γ treatment. These results clearly support a role for IL-32γ in promoting OB differentiation. We next investigated possible regulators that are related to the IL-32γ pathway at the mRNA level. The results revealed the IL-32γ induced suppression of *DKK-1*, a specific inhibitor of Wnt/β-catenin signaling that is secreted by OBs. Specifically, the mRNA levels of this protein were dramatically lower in the calvarial cells (Fig. 3b), and there was a decrease in the DKK-1 protein level in culture media after IL-32γ treatment (Fig. 3c). These results suggested that IL-32γ might regulate mouse *DKK-1* expression. To further validate the mechanism by which IL-32γ enhances OB differentiation in humans, we also determined the abundances of *DKK-1* mRNAs in human OBs and found the stimulation with IL-32γ reduced the abundance of *DKK-1* mRNAs (Fig. 3d). To confirm the downstream effects of *DKK-1* suppression, the level of active β-catenin was evaluated after the treatment with IL-32γ or Wnt3a (positive control). As shown in Fig. 3e, IL-32γ enhanced β-catenin activation significantly at 48 h, which could be related to suppression of *DKK-1*.

### Enhanced osteogenic differentiation in *IL-32γ* transgenic mice

**Fig. 4:**
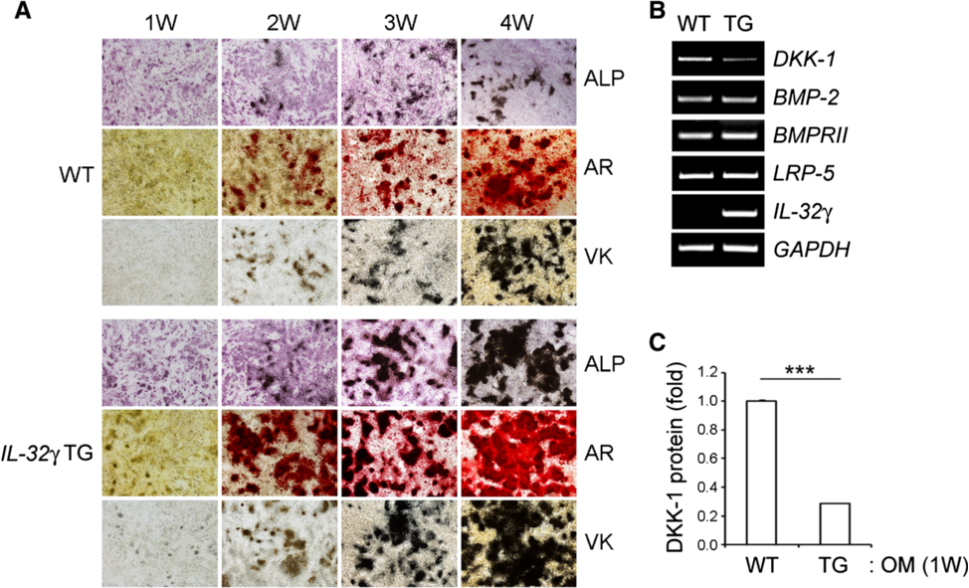
Osteogenic differentiation in *IL-32γ* TG mice. **a** Calvarial cells isolated from wild-type (*WT*) or *IL-32γ* transgenic (*TG*) mice were cultured in osteogenic medium (*OM*). The cells were fixed and used for the measurement of alkaline phosphatase (*ALP*) activity or for staining with alizarin red (*AR*), or Von Kossa (*VK*) stains to assess the degree of OB differentiation and mineralization at 1, 2, 3, and 4 weeks (*W*). **b** WT or *IL-32γ* TG calvarial cells were stimulated with osteogenic media for 24 h and subjected to reverse transcription PCR analysis of the expression of regulatory genes needed for OB differentiation, including those that encode *DKK-1*, *BMP-2*, *BMPRII*, and *LRP-5*. **c** The DKK-1 protein level in the culture supernatant from the cells after 1 week of OB differentiation was determined by ELISA. Representative data from at least three independent experiments are shown; ***p < 0.001. *BMP* bone morphogenetic protein, *BMPRII* bone morphogenetic protein receptor II, *DKK-1* Dickkopf-1, *IL* interleukin, *LRP-5* low-density lipoprotein receptor-related protein 5

Given the increased bone volume observed in *IL-32γ* TG mice, we next determined the role of OB differentiation in this phenomenon. The levels of ALP specific activity, calcium deposition, and mineral deposition indicated higher rates of osteogenic differentiation in *IL-32γ* TG mice than in WT mice (Fig. 4a). Reverse transcription PCR analysis revealed similar levels of the transcripts of genes that regulate OB differentiation—including *BMP-2*, *BMPRII*, and *LRP-5*—in the calvarial cells of the TG mice and WT mice after 24 h culture in osteogenic media. Interestingly, similarly to the endogenous reduction in *DKK-1* expression in the OB precursor cells induced by IL-32γ stimulation, the induction of *DKK-1* was dramatically lower in the calvarial cells from *IL-32γ* TG mice than those from WT mice (Fig. 4b). Furthermore, the DKK-1 protein level in the culture media from *IL-32γ* TG mice after 1 week of OB differentiation was also decreased (Fig. 4c). This finding suggests that the stimulation of osteogenic differentiation in *IL-32γ* TG mice might be linked to the reduction in the level of DKK-1 in the OB precursor cells.

## Discussion

The current study showed that the proinflammatory cytokine IL-32 is accumulated in inflamed joints in patients with AS more than in those with RA or OA that were selected as the comparison groups; RA is not a bone-forming disease but an inflammatory disease, whereas OA is less inflammatory but is a bone-forming disease. The IL-32 level in RA joints could be expected to be lower than that in OA when considering the aspect of bone formation. However, our findings revealing higher levels of IL-32 in RA joints than in OA joints are consistent with previous reports [12, 14]. We believe that the inflammatory cytokine IL-32 in RA joints could be accumulated by persistent inflammatory stimuli, although it was not enough to overcome bone loss and erosion. OA is characterized by osteophyte formation reflecting new bone formation, which is associated mainly with subchondral bone sclerosis, together with progressive cartilage damage [22], whereas new bone formation in AS involves a complicated and multifactorial sequence generated from the initiating entheseal inflammation.

Here, we verified the ability of IL-32γ to enhance OB differentiation and characterized how bone formation in AS differs from that in RA or OA. Our findings provide the first evidence, to our knowledge, of the high level of IL-32γ in AS peripheral and axial tissues and its association with abnormal bone formation. However, synovitis of peripheral joints could be a peripheral manifestation of disease, and does not correlate with axial joint severity and radiographic progression per se.

We demonstrated that *IL-32γ* TG mice had higher potency of osteogenic differentiation than WT mice and confirmed the enhanced OB differentiation triggered by the presence of the IL-32γ protein. An important aspect of IL-32 biology is that the induction of other pro-inflammatory cytokines, including TNF, IL-1, IL-18, and IL-22, has been suggested in human RA [23], indicating the possible involvement of pro-inflammatory cytokines in IL-32 function. However, it is intriguing that the *IL-32* TG mice, as well as IL-32 treatment, did not show any significant increases in the production of those cytokines under osteogenic stimulation conditions (Additional file 1). Interestingly, we found the reduction of IL-18 in *IL-32γ* TG mice and in cultures stimulated with IL-32, although this may not be significant. Unlike other inflammatory cytokines, IL-18 demonstrates positive effects on OB differentiation by mediating anabolic actions of parathyroid hormone [24]. In addition, IL-32 expression was correlated with IL-18 expression in the synovia of experimental arthritis animals and mucosa of patients with allergic rhinitis [25, 26]. However, we could not exclude the possibility that IL-32 and IL-18 regulate each other in a negative manner in OB differentiation. This assumption requires further investigation. Nevertheless, based on the knowledge of the inhibitory effects of pro-inflammatory cytokines (e.g., TNF, IL-1) on OB differentiation [27, 28], we can conclude that the osteogenic potential of IL-32 is unlike the activity of other pro-inflammatory cytokines.

A variety of chronic inflammatory diseases, including AS and RA, are currently treated by TNF inhibitors, especially in patients refractory to conventional treatments. Although TNF inhibitors are effective in controlling disease activity in AS, 2-year follow-up studies demonstrated no clear effect of this intervention on the development of bony ankylosis [29–31]. It was proposed that TNF suppresses OB differentiation by promoting DKK-1, which is a master negative regulator of the Wnt/β-catenin pathway [10]. Furthermore, a recent study revealed that a TNF inhibitor significantly decreased the level of serum DKK-1 in AS patients [32]. The emerging view is that TNF inhibits new bone formation during the inflammatory process, but that TNF inhibitors can restore OB function despite their anti-inflammatory effects.

However, a long-term observational study by Haroon et al., in which large numbers of patients were analyzed over a 5-year follow-up period, reported that after adjusting for the baseline mSASSS the AS patients who were administered TNF inhibitors showed a 50 % reduction in radiographic progression compared to those who had not received these agents [33]. To date, whether inflammation and new bone formation are uncoupled or occur simultaneously remains controversial. Therefore, considering the identified effects of IL-32γ on OBs and inflammation, treatments that target IL-32γ might prevent the uncoupling of inflammation from bony proliferation.

Although little is known about the mechanisms of syndesmophyte formation in the joints in AS, this likely involves regulatory molecules, such as Wnt proteins. Activation of Wnt signaling by blocking its natural inhibitor DKK-1 leads to the new bone formation in peripheral joints [10]. Interestingly, DKK-1 inhibition also leads to a bilateral erosive change and ankylosis of the sacroiliac joints in *TNF* TG mice, which have symptoms that mimic those of sacroiliitis in humans [34]. Given that DKK-1 is a key factor for joint remodeling during the inflammatory process, the AS phenotype might be related to certain conditions when DKK-1 has been suppressed or distorted functionally [35]. In this context, IL-32γ could be one of the important regulators that controls DKK-1 leading to modulation of the Wnt/β-catenin signal during the pathogenesis of AS.

Despite our interesting results, the limitations of this study include that ectopic bone formation in the joints, which can be affected by OB activation, did not develop in our TG model spontaneously. Moreover, because of small sample size the levels of IL-32 in the peripheral joints were not correlated significantly with several clinical parameters including mSASSS, which might be obstacles to link our findings to clinical research. Finally, we could not exclude the effect of age and gender on the IL-32 production clearly, although there was no association in our samples.

## Conclusions

Effective control of soluble factors that are related to bone proliferation might minimize disease progression in AS. Here, we show that AS joints have a higher level of IL-32γ, and the differentiation of OB precursors from *IL-32γ* TG mice into mature OBs might be related to the suppression of *DKK-1* expression. The higher levels of IL-32γ in the joints and tissues of the AS patients might induce OB differentiation and then trigger atypical new bone formation. These data suggest that IL-32γ might be a molecular target with the potential to prevent radiographic progression in AS.

## Additional file

Additional file 1:
**The protein levels of inflammatory cytokines including TNF-α, IL-1β, IL-18 and IL-22 were determined in the culture supernatant from the cells of WT or** ***IL-32γ*** **TG mice (A) and the cells in the absence (None) or presence of IL-32γ (B) after 1 week of OB differentiation using commercial available ELISA kits.** The bars show the means ± SD of triplicate experiments. (TIFF 443 kb)

## Abbreviations

ALP: Alkaline phosphatase
AR: Alizarin Red
AS: Ankylosing spondylitis
BMP: Bone morphogenetic protein
BMPRII: bone morphogenetic protein receptor II
DKK-1: Dickkopf-1
ELISA: Enzyme-linked immunosorbent assay
IHC: Immunohistochemical
IL: Interleukin
LRP-5: low density lipoprotein receptor-related protein 5
mSASSS: Modified Stoke Ankylosing Spondylitis Spine Score
OA: Osteoarthritis
OB: Osteoblast
PBS: Phosphate-buffered saline
PCR: Polymerase chain reaction
RA: Rheumatoid arthritis
TG: Transgenic
TNF: Tumor necrosis factor
VK: Von Kossa
WT: Wild-type
