# Supplementary figures and images for: High level of interleukin-32 gamma in the joint of ankylosing spondylitis is associated with osteoblast differentiation
Source: Arthritis Res Ther. 2015 Dec 4;17:350. doi: 10.1186/s13075-015-0870-4 (PMC4669668; doi:10.1186/s13075-015-0870-4)

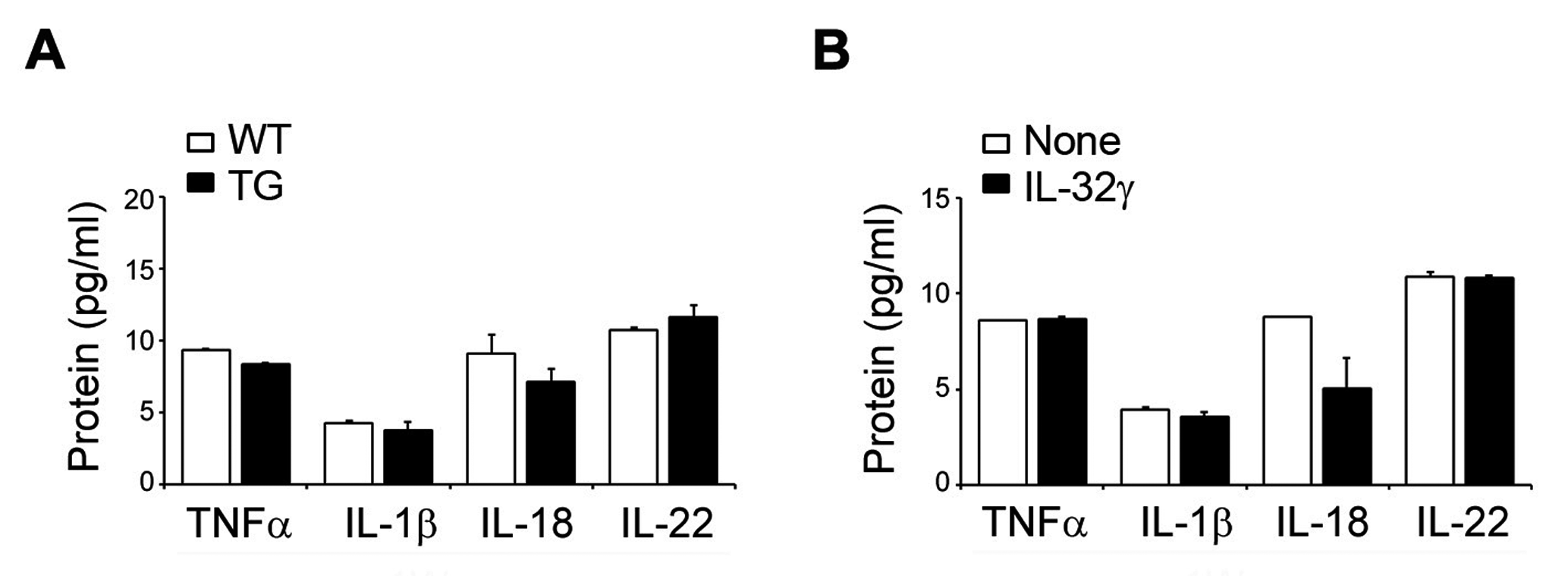

Supplement: Additional file 1: — The protein levels of inflammatory cytokines including TNF-α, IL-1β, IL-18 and IL-22 were determined in the culture supernatant from the cells of WT or IL-32γ TG mice (A) and the cells in the absence (None) or presence of IL-32γ (B) after 1 week of OB differentiation using commercial available ELISA kits. The bars show the means ± SD of triplicate experiments. (TIFF 443 kb) [file 13075_2015_870_MOESM1_ESM.tiff]
